# Murine models of scrub typhus associated with host control of *Orientia tsutsugamushi* infection

**DOI:** 10.1371/journal.pntd.0005453

**Published:** 2017-03-10

**Authors:** Nicole L. Mendell, Donald H. Bouyer, David H. Walker

**Affiliations:** Department of Pathology, Center for Biodefense and Emerging Infectious Diseases, Center for Tropical Diseases, Sealy Center for Vaccine Development, Institute of Human Infections and Immunity, School of Medicine, University of Texas Medical Branch, Galveston, Texas, United States of America; Johns Hopkins Bloomberg School of Public Health, UNITED STATES

## Abstract

**Background:**

Scrub typhus, a febrile illness of substantial incidence and mortality, is caused by infection with the obligately intracellular bacterium *Orientia tsutsugamushi*. It is estimated that there are more than one million cases annually transmitted by the parasitic larval stage of trombiculid mites in the Asia-Pacific region. The antigenic and genetic diversity of the multiple strains of *O*. *tsutsugamushi* hinders the advancement of laboratory diagnosis, development of long-lasting vaccine-induced protection, and interpretation of clinical infection. Despite the life-threatening severity of the illness in hundreds of thousands of cases annually, 85–93% of patients survive, often without anti-rickettsial treatment. To more completely understand the disease caused by *Orientia* infection, animal models which closely correlate with the clinical manifestations, target cells, organ involvement, and histopathologic lesions of human cases of scrub typhus should be employed. Previously, our laboratory has extensively characterized two relevant C57BL/6 mouse models using *O*. *tsutsugamushi* Karp strain: a route-specific intradermal model of infection and persistence and a hematogenously disseminated dose-dependent lethal model.

**Principal findings:**

To complement the lethal model, here we illustrate a sublethal model in the same mouse strain using the *O*. *tsutsugamushi* Gilliam strain, which resulted in dose-dependent severity of illness, weight loss, and systemic dissemination to endothelial cells of the microcirculation and mononuclear phagocytic cells. Histopathologic lesions included expansion of the pulmonary interstitium by inflammatory cell infiltrates and multifocal hepatic lesions with mononuclear cellular infiltrates, renal interstitial lymphohistiocytic inflammation, mild meningoencephalitis, and characteristic typhus nodules.

**Significance:**

These models parallel characteristics of human cases of scrub typhus, and will be used in concert to understand differences in severity which lead to lethality or host control of the infection and to address the explanation for short duration of heterologous immunity in *Orientia* infection.

## Author summary

Scrub typhus is an acute febrile illness with considerable mortality, and no available vaccine, caused by the obligately intracellular bacterium, *Orientia tsutsugamushi*. Despite the life-threatening severity of the illness in approximately one million cases annually, 85–93% of patients survive. The lack of appropriate animal models of scrub typhus has left a void in the fundamental knowledge necessary to develop a vaccine, such as mechanisms which contribute to disease severity and immunity. Here, we report a sublethal inbred murine model for scrub typhus using the intradermal and intravenous routes of inoculation, which are comparable to the natural route of chigger-bite transmission and subsequent hematogenous spread. This model, infection of mice with *O*. *tsutsugamushi* Gilliam strain, can be employed in conjunction with the lethal model of *O*. *tsutsugamushi* Karp strain to perform in-depth mechanistic studies related to strain cross-protection, lethality, pathogenesis and specific immunological investigations of the host immune response.

## Introduction

Scrub typhus is a potentially fatal febrile illness caused by infection with the obligately intracellular bacterium *Orientia tsutsugamushi*. The geographic range of confirmed cases includes Asia, islands of the Pacific and Indian Ocean, and northern Australia; areas home to more than one-third of the world’s population [1]. Moreover, growing evidence implicates a range of *Orientia* infection outside of the known endemic region, including a case transmitted in the United Arab Emirates, serological and molecular data from Africa and South America and molecular evidence which has suggested *Orientia* species are present in Europe [2,3,4,5,6,7,8,9,10]. Individuals are infected with the bacteria transmitted to humans during feeding by infected larval trombiculid mites. Foci of transmission correspond to the distribution of the chigger mite vectors whose habitat consists of secondary or transitional forms of vegetation that exist after environmental modification such as removal of primary forests, practice of shifting cultivation, abandonment of fields, plantations and village sites during conflict, and neglect of urban and suburban garden plots [11,12,13,14]. The prospect of increasing vector habitat and the wide geographic distribution stress the importance and widespread impact of this disease, emphasizing the need for an effective vaccine.

Scrub typhus presents one to two weeks after exposure with a not-always-observed bite-site eschar and regional lymphadenopathy, followed by fever and rash accompanied by non-specific flu-like symptoms, requiring empirical treatment based on presumptive etiology. If prompt and appropriate antibiotic therapy is not administered, multi-organ failure and death can follow [15,16,17,18,19,20,21,22]. Fatal scrub typhus is characterized by disseminated endothelial infection, diffuse interstitial pneumonia, hepatic lesions, acute renal failure, and meningoencephalitis [23,24,25,26,27,28,29,30,31]. In scrub typhus autopsy or eschar samples, *Orientia* have been observed intracellularly in endothelial cells, macrophages, dendritic cells, and cardiac myocytes [24,28,32]. Understanding the systemic immune and pathophysiological mechanisms of scrub typhus in humans early in the course or in non-fatal cases is limited by sample size, diagnostic acuity, and invasiveness of sampling. Employing an appropriate animal model, which produces disease severity, pathology and systemic endothelial infection resembling human infection, may be used to overcome this impediment to understanding scrub typhus disease progression and the host immune mechanisms necessary for effective vaccine development.

The adaptive immune response against *O*. *tsutsugamushi* is not well characterized, is short-lived, complicated by strain diversity, and does not afford sterile protection. Studies of naturally acquired *O*. *tsutsugamushi* and vaccine studies in humans using live organisms have provided evidence of strain-specific protection, persistent infection with the immunizing strain, short-lived heterologous immunity, and simultaneous infection with multiple strains of *Orientia* [33,34,35,36,37,38,39,40,41,42,43]. Numerous animal models have shown that inoculation with live-, fixed-, or replication-deficient-*O*. *tsutsugamushi* have afforded partial, time-dependent protection against the homologous strain and poor, waning protection against heterologous strains of *O*. *tsutsugamushi* [44,45,46,47,48,49,50,51]. The mouse models used for these protection studies have employed intraperitoneal challenge, resulting in severe and lethal peritoneal infection and inflammation, which are not a characteristic of natural infection [52,53,54,55]. Although the data from previous murine studies confer valuable information, conclusions about the events of long-term, cell-mediated immunity against *O*. *tsutsugamushi* still need to be elucidated from more appropriate animal models. This well-characterized model of *O*. *tsutsugamushi* Gilliam strain infection is a necessary addition to the murine model repertoire for future studies of immunity to scrub typhus.

Herein, we report the dose- and route-specific kinetics of bacterial dissemination and disease progression of a model of sublethal scrub typhus utilizing the *O*. *tsutsugamushi* Gilliam strain. Recent advances to utilizing inbred murine models with more relevant routes and characterization of the pathogenic features of human scrub typhus have been achieved using the Karp strain [55,56,57]. This sublethal model of disseminated Gilliam strain infection is a crucial addition to research efforts to understand host-pathogen interactions influencing sublethal versus lethal outcomes and heterologous strain immunity dynamics.

## Materials and methods

### Ethics statement

All experiments and procedures were approved by the Institutional Animal Care and Use Committee (protocol # 1302003) of the University of Texas Medical Branch-Galveston. Mice were used according to the guidelines in the Guide for the Care and Use of Laboratory Animals and comply with the USDA Animal Welfare Act (Public Law 89–544), the Health Research Extension Act of 1985 (Public Law 99–158), the Public Health Service Policy on Humane Care and Use of Laboratory Animals, and the NAS Guide for the Care and Use of Laboratory Animals (ISBN-13).

### Cell culture

L929 and Vero cells (ATCC, Manassas VA) were maintained in Dulbecco’s Modified Eagle Medium (DMEM, Gibco Life Technologies, Grand Island, NY) supplemented with 5% fetal bovine serum (FBS, HyClone Laboratories, Logan UT) and 1% HEPES buffer (Cellgro, Manassas, VA) at 37°C with 5% CO_2_ in a humidified incubator.

### Stock propagation

*Orientia tsutsugamushi* Gilliam strain (unknown passage history) was obtained from the Rickettsial and Ehrlichial Species Collection at the University of Texas Medical Branch. Identification of the strain was confirmed by sequencing of the *Orientia* 47 kDa gene (accession number L31933). *Orientia* was propagated 3 passages in L929 cells from yolk sac seed stock and stored at -80°C in sucrose-phosphate-glutamate (SPG) buffer (218 mM sucrose, 3.8 mM KH_2_PO_4_, 7.1 mM K_2_HPO_4_, 4.9 mM monosodium L-glutamic acid, pH 7.0) until further use.

### Viability assay and bacterial load determination

An *Orientia* quantitative viability assay was utilized to enumerate viable *Orientia* as previously described [56]. Briefly, confluent 6-well plates of Vero cells were inoculated in triplicate with serial 10-fold dilutions of *Orientia* stocks prepared in Dulbecco’s phosphate buffered saline (DPBS, Cellgro, Manassas, VA). The plates were centrifuged for 5 minutes at 700 x *g* to enhance oriential contact with cells and incubated for two hours at 34°C with 5% CO_2_. After two hours, the wells were triple rinsed with warm DPBS with calcium and magnesium to remove extracellular bacteria. DNA was extracted from each well using a DNeasy Blood and Tissue Kit (Qiagen, Valencia, CA) according to the manufacturer’s instructions, and the bacterial load determined by quantitative real-time PCR (qPCR) to determine the quantity of viable *Orientia* that had attached and actively entered Vero cells.

The single copy gene for the 56-kDa protein was amplified with primers [OtG729 (5′- TCGTGATGTGGGGGTTGATAC-3′) and OtG873 (5′- ATTCTGAGGATCTGGGACCATATAG-3′) (IDT, Coralville, IA)] to determine *Orientia* copy numbers. qPCR was accomplished using iQ SYBR-green supermix (Bio-Rad, Hercules CA) with a Bio-Rad CFX96 thermal cycler according to the protocol: one cycle at 94°C for 5 minutes followed by 40 cycles of two-step amplification at 94°C for 5 seconds and 61.8°C for 30 seconds. Serial 10-fold dilutions of a known concentration of a plasmid that contained a single copy of the 56-kDa gene were utilized to produce a standard curve to determine copy numbers. Bacterial loads and dissemination to selected organs were assessed by qPCR. DNA was extracted using a DNeasy Blood and Tissue Kit (Qiagen, Valencia, CA) from bead homogenized tissue samples according to the manufacturer’s instructions. Tissues samples were normalized using tissue wet weight and were expressed as the number of *O*. *tsutsugamushi* Gilliam strain 56 kDa copies per milligram (mg) of tissue.

### Mouse infection

Female C57BL/6 (B6) mice, 6–8 weeks of age, purchased from Harlan Laboratories (Indianapolis, IN) were housed in an animal biosafety level 3 facility (ABSL3) under specific pathogen-free conditions. The mice were allowed to acclimate for 7 days prior to experimental use and then were inoculated i.v. by the tail-vein with 3 doses: high (7.5 x 10^6^), mid (7.5 x 10^5^), or low (7.5 x 10^3^) or intradermally in the lateral ear with 2.5 x 10^5^ *O*. *tsutsugamushi* organisms as determined by viability assay and monitored twice daily for signs of illness. For the i.v. infected animals, one group (N = 5) of mice from each dose was sacrificed every three days for a period of fifteen days, and one group (N = 5) of i.d. inoculated mice was sacrificed every six days for a period of 30 days. Mice were necropsied, and their tissues were tested for bacterial burden and prepared for histology. The remaining animals were observed for veterinary-approved signs of illness (ruffled fur, hunched posture, erythema, lethargy, conjunctivitis, and weight loss). All animal experiments were conducted twice.

### Hematologic analyses

At the designated sacrifice time points, blood samples (500 μL) were collected in K_2_EDTA-coated BD microtainer tubes (Becton, Dickinson and Company, Franklin Lakes, NJ) and blood cell counts performed using a calibrated 950FS HemaVet apparatus (Drew Scientific, Waterbury CT). The blood samples were analyzed using the FS-Pak reagent kit and were measured for the following parameters: white blood cell count (WBC), differential leukocyte (%) count, hemoglobin concentration (HGB), hematocrit (HCT), red blood cell count (RBC), mean corpuscular volume (MCV), mean corpuscular hemoglobin (MCH), mean corpuscular hemoglobin concentration (MCHC), red cell distribution width (RDW), platelet count (PLT), and mean platelet volume (MPV).

### Indirect immunofluorescence assay

*Orientia tsutsugamushi* Gilliam strain antigen-coated, acetone-permeabilized 12-well slides were equilibrated to room temperature in phosphate buffered saline (PBS) and then blocked in PBS containing 1% bovine serum albumin (BSA) and 0.01% sodium azide for 10 minutes at room temperature. Sera were diluted 2-fold starting at 1:64 and, if reactive, extended to final end-point titers in a solution of PBS containing 1% BSA, 0.1% Tween 20, and 0.01% sodium azide. Dilutions of sera were added to individual antigen-coated wells and incubated at 37°C for 30 minutes in a humidified chamber. Slides were rinsed and washed twice for 10 minutes with PBS containing 0.1% Tween-20. Secondary antibody, either DyLight 488-conjugated anti-mouse IgG (1:15000), Fluorescein (FITC)-conjugated AffiniPure anti-mouse IgG Fcγ subclass 1specific (1:600), FITC-conjugated AffiniPure anti-mouse IgG, Fcγ subclass 2c specific (1:1000, Jackson Immunoresearch, West Grove, PA) or FITC-conjugated anti-mouse IgM antibody, mu chain specific (1:500, Vector Labs, Burlingame, CA), was incubated for 30 minutes at 37°C in a humidified chamber. Slides were subsequently rinsed and washed twice as before with the final wash containing 1% Evans blue solution, mounted with DAPI fluoromount-G (SouthernBiotech, Birmingham, AL) and coverslipped. Slides were observed under a fluorescence microscope at 400X magnification (Olympus Scientific, Waltham, MA). Serum was unavailable for one mouse from the i.d. route group on 18 dpi (n = 4), otherwise n = 5 for all groups and time points. Mice that had a positive IFA result at a 1:64 dilution were considered to have seroconverted, whereas mice with non-reactive serum at this titer were assigned a value of zero.

### Histology

Tissue samples were fixed in 10% neutral buffered formalin (NBF) and embedded in paraffin. Tissue sections (5 μm thickness) were stained with hematoxylin and eosin or processed for immunohistochemistry (IHC). For IHC, all reagents were from Vector Laboratories (Burlingame, CA) unless specified otherwise. Slides were deparaffinized, rehydrated and processed. Antigen retrieval was performed by incubation in 1x citrate buffer (Labvision, Fremont, CA). Sections were sequentially blocked with 1x casein, BLOXALL blocking solution, avidin and biotin solution and 5% normal goat serum. Sections were incubated with polyclonal rabbit anti-*O*. *tsutsugamushi* antibody (1:12000, produced in-house) at 4°C overnight, followed by incubation with biotinylated anti-rabbit IgG (1:200) for 30 minutes. Signals were developed with Vector Red Alkaline Phosphatase substrate kit. Slides were counterstained with hematoxylin, dehydrated, mounted and cover slipped with VectaMount and examined with an Olympus BX51 microscope (Olympus Scientific, Waltham, MA).

### Analysis of histopathology

Sections were examined to assess the histopathology and establish grading scales. The slides were then examined blindly, without knowledge of dpi or bacterial loads, and scores were determined independently based on the grading systems described below. The grading scale for the lung histopathology was based on the spectrum of lesions throughout the entire course of infection (S1 Fig). Grade 1 was defined as scattered inflammatory cells in focal areas of pulmonary parenchyma and around bronchovascular bundles. A score of 2 was assigned to tissues with widening of alveolar septa and inflammatory cell infiltrates present multifocally in the pulmonary parenchyma and around bronchovascular bundles. Grade 3 was defined as similar to grade 2 but present more diffusely in the pulmonary parenchyma and around bronchovascular bundles and Grade 4 was assigned to tissues presenting with the description of Grade 3 plus areas of atelectasis. The diameters of hepatic clusters of inflammatory infiltrates were measured, and the average lesion size and number of lesions per four typical (100X) fields of liver were determined for each time point. The liver inflammatory index was calculated as number of lesions per four medium-power fields (MPF) multiplied by the mean diameter (μm) of mononuclear cellular infiltrative clusters. Quantitative assessment of the renal histopathology was based on the extent of mononuclear cellular infiltrate. Digital images of four to six randomly selected medium power fields (100X) of the renal cortex were captured using Olympus DP controller software. Semi-automated counting was performed using ImageJ (National Institutes of Health, Bethesda, MD, USA) after converting the image to 8-bit grayscale. Cells contributing to the total mononuclear cell count were identified using a manual threshold and pixel-based area measurement. The number of pixels was counted and presented as a proportion of the total number of pixels in the area under analysis.

### Statistical analysis

Values are reported as mean ± standard deviation (SD). The data were analyzed using an one-way ANOVA with Tukey’s multiple comparison as post-hoc analysis (GraphPad Prism, San Diego, CA) at a statistical significance level of *, p < 0.05; **, p < 0.01, ***, p < 0.001.

## Results

### Clinical signs of infection with *O*. *tsutsugamushi* Gilliam strain

**Fig 1:**
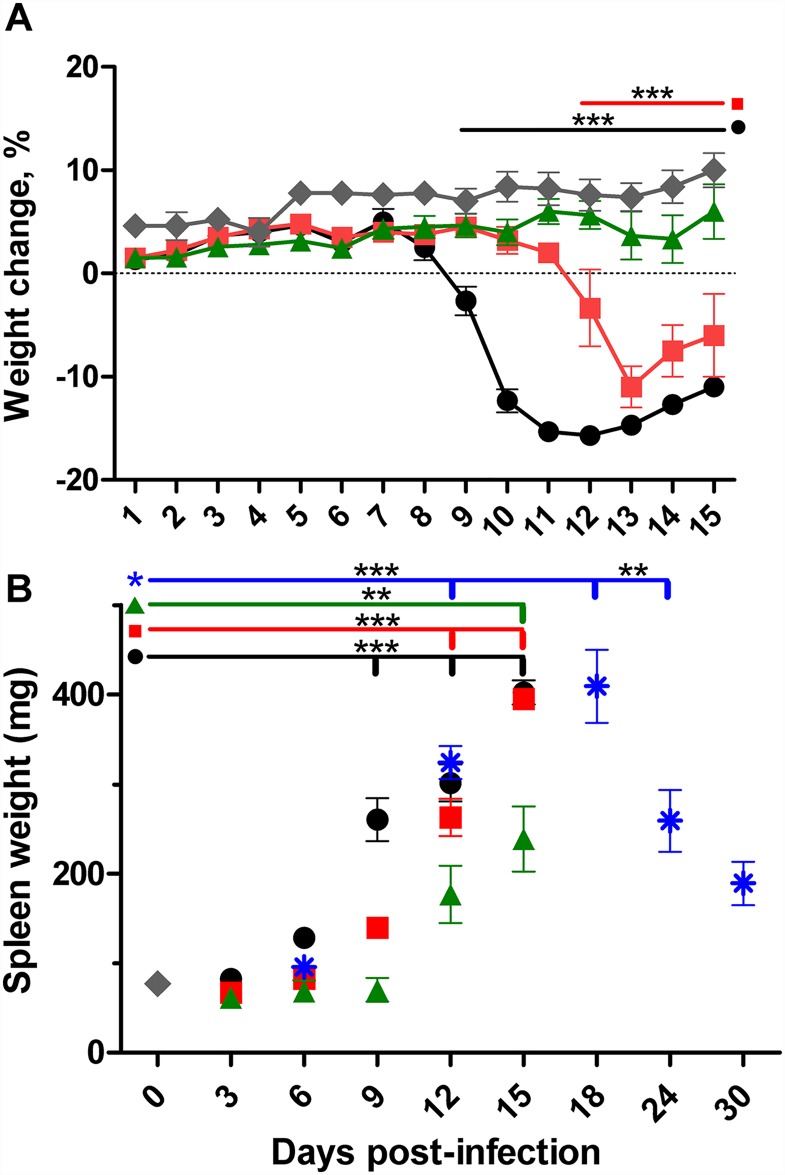
Body and spleen weight change of mice inoculated intravenously or intradermally with *O*. *tsutsugamushi* Gilliam strain. Percent body weight change (**A**) or spleen weight in milligrams (**B**) of animals inoculated intravenously with either 7.5x10^6^ (high dose, **circles**), 7.5x10^5^ (mid-dose, **squares**), 7.5x10^3^ (low dose, **triangles**), or intradermally with 2.5x10^5^ organisms (**asterisks**) as compared to sham inoculated control (**diamonds**). **, *p*<0.01, ***, *p*<0.001.

The dose responses of C57BL/6 mice to intravenous inoculation with *O*. *tsutsugamushi* Gilliam strain were observed as differences in incubation period prior to onset, duration of illness, magnitude of signs of illness and percent body weight loss. Mice infected intravenously (i.v.) with a high dose of *O*. *tsutsugamushi* Gilliam strain developed decreased activity (7–12 dpi), ruffled fur and erythema (6–12 dpi), labored breathing (9–11 dpi), conjunctivitis (11–12 dpi) and began to lose weight at day 8 pi, with a nadir mean percent body weight (16% loss) by day 12 pi (Fig 1A). The animals inoculated i.v. with the mid-dose developed signs of illness including decreased activity, ruffled fur labored, breathing and skin erythema on 10–12 dpi followed by weight loss delayed by 3 days with mean nadir percent body weight loss (11%) observed at 13 dpi. The mean weights for the group receiving the low dose i.v. did not decrease below the mean starting weight; however, it was below the mean weight of the uninfected controls at 14 dpi followed by perceptible labored breathing at 15 dpi. After intradermal inoculation, mice were monitored through 30 dpi, during which mean percent body weight did not deviate below uninfected controls. However, decreased activity was observed from 12–16 dpi, ruffled fur during 13–18 dpi, and conjunctivitis on 13 dpi. Onset and duration of splenomegaly, significant increase of whole spleen wet weight above uninfected controls, was observed in a dose- and route-dependent manner. Splenomegaly was observed in mice inoculated i.v. with the high dose from 9 dpi until the end of study of these animals on day 15, in mice inoculated i.v. with the mid-dose from 12 dpi to the end of the observations on day 15, and in mice inoculated i.v. with the low dose at 15 dpi (Fig 1B). Intradermal inoculation resulted in splenomegaly during 12–24 dpi, with the peak at 18 dpi comparable to that of high dose i.v. inoculation.

### Hematologic responses to sublethal infection with *O*. *tsutsugamushi* Gilliam strain

**Fig 2:**
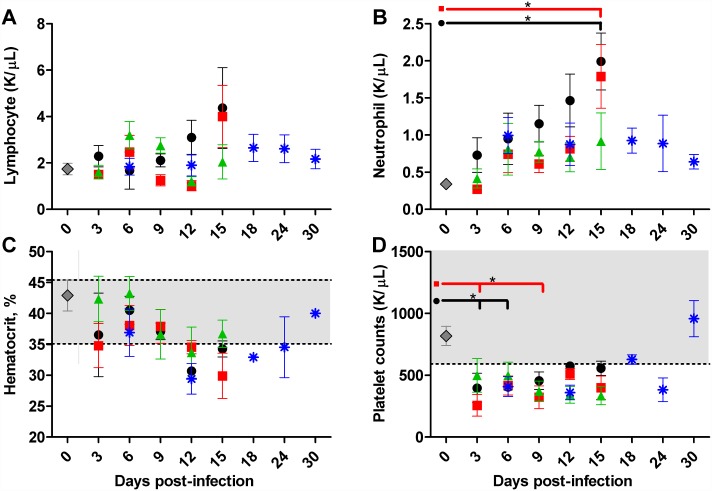
Hematologic responses to *O*. *tsutsugamushi* challenge. Circulating absolute lymphocyte (**A**) and neutrophil concentrations (**B**), percent hematocrit (**C**) and platelet counts (**D**) of uninfected (n = 5, **diamonds**) mice or those inoculated (n = 5) intravenously with high dose (**circles**) mid-dose (**squares**) low dose (**triangles**) or via intradermal route (**asterisks**) Grey shading represents murine normal range. *, *p*<0.05.

Peripheral blood parameters of infected mice were compared to uninfected controls and veterinary accepted normal ranges for mice. While circulating lymphocyte counts remained within the normal range, the mean lymphocyte count for mice infected with high dose i.v. was elevated above the mean of uninfected controls (1.74 K/μL) at 12–15 dpi (3.09–4.37 K/μL) and was elevated on 15 dpi (4.01 K/μL) for i.v. mid-dose (Fig 2A). A steady mean increase in absolute neutrophil concentrations was observed during 9–15 dpi for high dose i.v. infected mice, reaching statistical significance by 15 dpi (Fig 2B). A significant increase in neutrophil concentration was also observed at 15 dpi in mice infected with the mid-dose i.v. Less consistent elevation of neutrophils occurred after low dose i.v. inoculation or after i.d. inoculation. Decreases in hematocrit and platelet count below the normal range were observed regardless of dose or route of inoculation (Fig 2C and 2D).

**Fig 3:**
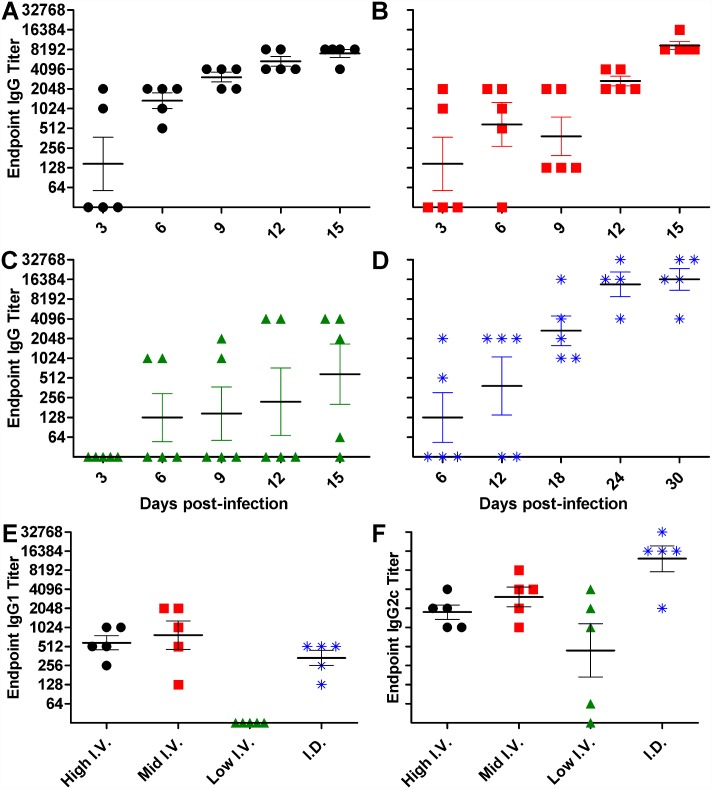
Seroconversion after infection with *O*. *tsutsugamushi* Gilliam strain. Reciprocal endpoint IgG titer of mice as determined by indirect IFA after i.v. inoculation with high (**A**), mid (**B**), or low dose (**C**) or via the intradermal route (**D**). The IgG isotype response was further categorized into IgG1 (**E)** or IgG2c (**F**) at the final time point, 15 dpi for the i.v. route and 30 dpi for the i.d. route. Serum which was nonreactive at a 1:64 titer is represented by a value of zero.

Seroconversion was first observed at day 3 after high dose (Fig 3A) i.v. inoculation with 80% and 40% (n = 5) having IgM and IgG anti-*O*. *tsutsugamushi* antibody reactivity, respectively, followed by 100% IgM and IgG seroconversion on 6 (Table 1, Fig 3A). Mid-dose i.v. infection induced 80% IgM and 40% IgG seroconversion by 3 dpi followed by 100% seroconversion at 6 dpi and 9 dpi for IgM and IgG, respectively (Table 1, Fig 3B). The mice which received low dose i.v. inoculation (Fig 3C) had seroconversion of 40% IgM at 3 dpi, followed by 40% of animals seroreactive for IgM and IgG at 6 dpi, and while 100% had IgM antibodies, only 80% had IgG seroconversion by 15 dpi, the last experimental time point (Table 1, Fig 3C). Animals infected by intradermal inoculation had IgM seroconversion in 80% of mice and IgG seroconversion in 40% of mice at 6 dpi with all mice seroconverted by 18 dpi and a continual increase in antibody titer through the final time point, 30 dpi (Table 1, Fig 3D). At the final experimental time point, the reciprocal endpoint titer of IgG2c antibodies was higher than IgG1 for high dose i.v. (medians 2048 vs. 512), mid-dose i.v. (medians 4096 vs. 1024), low dose i.v. (medians 1024 vs. 0), and i.d. route (medians 16384 vs. 512) (Fig 3E and 3F).

**Table 1:** Development of antibodies to *O*. *tsutsugamushi* Gilliam strain following challenge.

	d3		d6		d9		d12		d15		d18		d30	
	IgM	IgG	IgM	IgG	IgM	IgG	IgM	IgG	IgM	IgG	IgM	IgG	IgM	IgG
**High dose I.V.**	80	40	100	100	100	100	100	100	100	100				
**Mid dose I.V.**	60	40	100	80	100	100	100	100	100	100				
**Low dose I.V.**	40	0	40	40	80	40	100	40	100	80				
**I.D.**			80	40			100	60			100	100	100	100

Percentage of mice that seroconverted following challenge as determined by indirect immunofluorescence assay (IFA). None of the control mice were seropositive at the experimental cutoff of 1:64 dilution.

### Bacterial dissemination in mice infected with *O*. *tsutsugamushi* Gilliam strain

**Fig 4:**
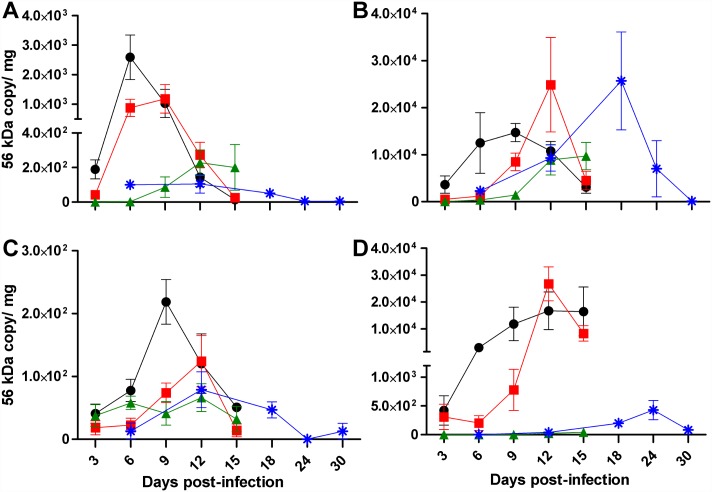
Bacterial dissemination after infection with *O*. *tsutsugamushi* Gilliam strain. Bacterial loads in spleen (**A**), lung (**B**), liver (**C**) and kidney (**D**) after i.v. inoculation with high (**circles**), mid (**squares)**, or low dose (**triangles**), or via i.d. inoculation **(asterisks)**.

Intravenous inoculation resulted in dose-dependent, self-limited systemic infection. The earliest peak of bacterial burden after i.v. inoculation was observed in the spleen on 6 dpi after high dose, 9 dpi after mid-dose, and from 12–15 dpi after low dose inoculation (Fig 4A). Of the tissues examined, the lungs had the highest bacterial load starting at 3 dpi and reached a peak at 9 dpi for the high dose i.v. group (Fig 4B), which was the day of onset of weight loss (Fig 1A). The peak of bacterial load for the i.v. mid-dose recipient mice was observed later, at 12 dpi (Fig 4B). In contrast, a sustained peak was observed at 12 and 15 dpi in the i.v. low dose inoculated mice. The same trend was observed for hepatic bacterial loads but with a lower bacterial load per milligram of tissue (Fig 4C). Intravenous inoculation resulted in higher bacterial loads in the kidney than i.d. inoculation (Fig 4D). A steady increase of bacterial loads was observed in the kidney after i.v. high and mid-dose inoculation, reaching a sustained peak at 9–15 dpi with the high dose and a peak at 12 dpi with the mid-dose. Renal bacterial loads after i.v. low dose inoculation were detected inconsistently.

### Histopathology and target cells as determined by IHC

**Fig 5:**
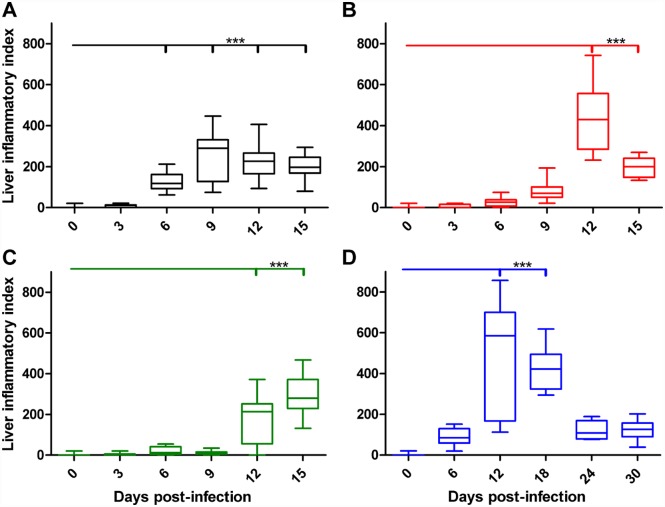
Dose-dependent liver inflammatory index of mice inoculated i.v. or i.d. with *O*. *tsutsugamushi* Gilliam strain. Liver inflammatory index after i.v. inoculation with high (**A**), mid (**B**) or low (**C**) dose or i.d. (**D**) with *O*. *tsutsugamushi*. ***, *p*<0.001.

The histopathology of the liver in this model was characterized by multifocal oriential vascular infection and persistent multifocal lesions typified by multifocal lymphohistiocytic and polymorphonuclear cellular infiltrates, vasculitis, and dose-dependent portal triaditis (S2C Fig). Initially, the magnitude of lymphohistiocytic cellular infiltration coincided with increased bacterial loads; however, it continued to intensify as the bacterial loads were controlled. Intravenous high dose inoculation resulted in periportal lymphohistiocytic infiltrates at 3 dpi and focal lesions in the sinusoids at 6 dpi. The lobular lesions were more numerous and encompassed greater tissue area by 6 dpi, as indicated by the increased liver inflammatory index, and were unresolved at the experimental endpoint of 15 dpi (Fig 5A). The peak liver inflammatory index occurred at 12–15 dpi for mid and low dose i.v. route (Fig 5B and 5C). Following i.d. inoculation, the peak liver inflammatory index occurred at 12–18 dpi, and although the lesions decreased in diameter and quantity, they were still present at the experimental endpoint of 30 dpi (Fig 5D).

**Fig 6:**
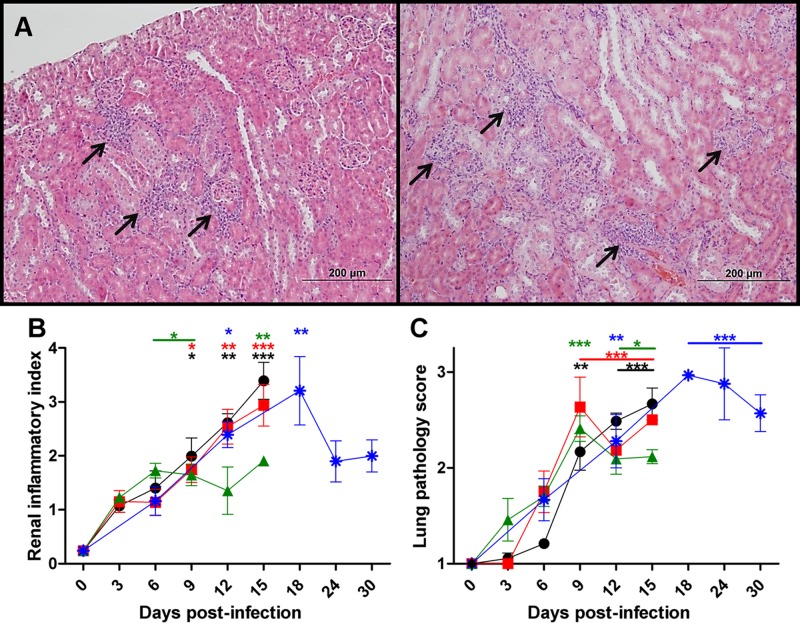
Dose-dependent severity of histopathologic renal lesions and lung pathology in mice inoculated i.v. or i.d. with *O*. *tsutsugamushi* Gilliam strain. Representative histopathologic renal inflammatory infiltrates between tubules of the renal cortex (arrows) at 6 dpi after i.d. inoculation (**A**, left) and i.v. mid-dose at 15 dpi (**A**, right, 100X). Renal inflammatory index (**B**) or lung pathology score (**C**) after i.v. inoculation with high (**circles**), mid (**squares**) low (**triangles**) dose or i.d. (**asterisks**) with *O*. *tsutsugamushi*. *, *p*<0.05, **, *p*<0.01, ***, *p*<0.001. Asterisks with bars indicate data points statistically different from baseline.

Nephritis, characterized by interstitial cellular infiltrates in the renal cortical parenchyma, which were localized between renal tubules and commonly at the corticomedullary boundary, was observed in the kidneys of mice inoculated with *O*. *tsutsugamushi* Gilliam strain regardless of route or dose of inoculum (Fig 6A). The kidney inflammatory ratio continued to intensify through the experimental endpoint, 15 dpi after i.v. high and mid-dose inoculation (Fig 6B). After i.v. low dose inoculation, the kidney inflammatory ratio plateaued at 6 dpi and remained elevated through 15 dpi. The peak of the kidney inflammatory ratio after i.d. inoculation occurred at 18 dpi and was unresolved at day 30 pi.

Lung pathology during *O*. *tsutsugamushi* Gilliam strain infection progressed throughout the time course observed, and the onset of significant pathology scores correlated with the route of inoculation. Mild, isolated peribronchovascular inflammation was observed at 3 dpi in i.v. high dose infected mice followed by widening of the alveolar septa by 6 dpi. Beginning at 9 dpi patchy frank interstitial cellular inflammation was observed with mild vasculitis. At subsequent time points after high dose i.v. inoculation, from 9 dpi through day 15 pi, peribronchial infiltrates continued to be evident, and the interstitial inflammation was more diffuse and encompassed larger portions of the tissue. After mid-dose inoculation, the peak of pulmonary inflammation occurred at 9–15 dpi and was significantly elevated, and greater than after i.v. low dose inoculation at those time points. Pulmonary inflammation after i.d. inoculation was statistically significant at 12 dpi with the peak lung pathology score observed at 18–30 dpi (Fig 6C).

**Fig 7:**
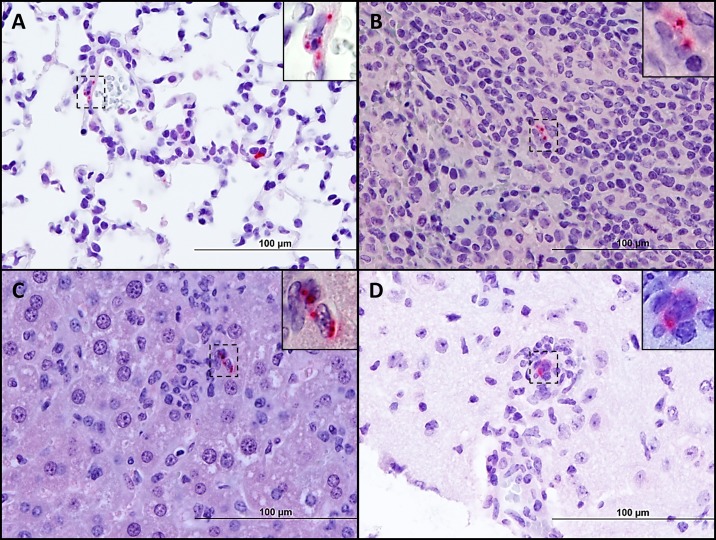
Location *of O*. *tsutsugamushi* Gilliam antigens following i.v. or i.d. inoculation. Sections of the lungs at 9 dpi after i.v. inoculation reveal the presence of *Orientia* antigens (red) in interstitial capillary vessels and alveolar septa (**A**). Orientia antigens co-localize with splenic (**B**) and hepatic (**C**) macrophages. Orientia antigen in a cerebral vessel surrounded by a characteristic typhus nodule 18 dpi after i.d. inoculation (**D**, 400X, inset 1,000X, bars = 100 μm).

Mild myocarditis was observed after infection (S2A Fig). Mononuclear cellular infiltrate was observed in the pericardium and between cardiac myocytes. Mild meningoencephalitis and characteristic typhus nodules (clusters of perivascular microglial cells, macrophages and lymphocytes) were observed in mice inoculated both i.v. and i.d. (Fig 7D, S2D Fig). Expansion of the splenic marginal zone and lymphoid activation in periarteriolar lymphoid sheaths were observed after Gilliam strain inoculation (S2B Fig).

*Orientia* identified by IHC on 9 dpi, the peak of bacterial burden after high dose i.v. inoculation, was located in endothelial cells of the alveolar capillaries (Fig 7A), and in splenic and hepatic mononuclear phagocytes (Fig 7B and 7C). *Orientia* antigen was also observed in the endothelial cells of cardiac microcapillaries between bundles of cardiac myocytes. At the peak of bacterial load after i.d. inoculation at 18 dpi, *Orientia* antigen was identified in pulmonary and renal endothelial cells as well as in characteristic typhus nodules (Fig 7D).

## Discussion

Several mouse models of *O*. *tsutsugamushi* infection have been developed, but few are extensively characterized in an inbred mouse model to allow for elucidation of the mechanisms of pathogenesis and immunity correlating with human infection [58,59,60]. The use of inbred C57BL/6 mice to characterize *O*. *tsutsugamushi* infection will allow for consistent animal-to-animal responses required for the further testing of hypotheses and vaccine evaluation to a level of statistical significance necessary for interpretation of results. Utilizing the abundance of conditional and gene knockout mice available on this background will enable elucidation of the mechanisms of immunity to *O*. *tsutsugamushi*. Studies exploring inbred mouse susceptibility to *O*. *tsutsugamushi* Gilliam strain have reported resistance of C57BL/6 and C57BL/6J to high inoculum i.p. challenges [61,62]. We have characterized, in more detail, scrub typhus animal models employing the more relevant i.v. and i.d. routes of inoculation of *O*. *tsutsugamushi* Gilliam strain in C57BL/6 mice with hematogenous dissemination to pulmonary and systemic endothelial cells of the microcirculation, which results in disseminated self-limited disease mimicking the mite-transmitted infection in many persons. This mouse strain, in contrast, succumbs to challenge with *O*. *tsutsugamushi* Karp strain inoculated i.v. and i.p [55,61]. Lethal and sublethal endothelial cell target models using i.v. inoculation or i.d. inoculation, respectively, of *O*. *tsutsugamushi* Karp strain in C57BL/6 mouse strain have already been extensively characterized [55,56]. These well characterized scrub typhus animal models with divergent lethality utilizing the same inbred mouse strain may be employed for studies on virulence mechanisms of the bacterial strains and the protective host immune mechanisms. Although more than 70 strains of *O*. *tsutsugamushi* genetic variants have been identified, strains genetically related to the Karp and Gilliam strains have been reported with overlapping geographical distribution and are implicated as prevalent types causing scrub typhus illness, important considerations for designing experiments relevant to human disease and immunity [63].

To model scrub typhus, it would be ideal to study bacterial dissemination characteristics and host-pathogen interactions after natural chigger-bite infection. However, limited access to the infected *Leptotrombidium* vector colonies, the burden of colony maintenance, and inability to standardize the dose of bacterial transmission by the vector favors the utility of needle-inoculated animal models. Intradermal inoculation is the needle inoculation route which most closely mimics the natural vector transmission during cutaneous feeding. The i.v. route of infection results in a hematogenously disseminated systemic infection as occurs in human scrub typhus, but bypasses the events of early cutaneous infection and the initial spreading steps. The kinetics of infection following the i.v. and i.d. administration routes allows for optimized experimental design focusing on critical disease course time points. In contrast to what has been reported after subcutaneous footpad inoculation of *O*. *tsutsugamushi* Karp strain, characteristics of infection of i.v. and i.d. infection in these models including bacteremia, target organs and infected cell types are analogous with only varying kinetics [57,64].

In these models, we characterized the clinical signs including splenomegaly and the degree of weight loss, as well as kinetics of bacterial spread. We also analyzed the hematologic response to infection, rate of IgM and IgG antibody production, and antibody isotype. The histopathologic events in response to Gilliam strain challenge included development of interstitial pneumonitis, interstitial nephritis, mild meningoencephalitis, cerebral typhus nodules, and perivascular lymphohistiocytic inflammation in the lung, liver, heart, and kidney. Statistically significant lung inflammation was observed concurrently irrespective of dose or route and was sustained after the peak of infection, suggesting elicitation by a host-mediated inflammatory response. This model recapitulates the pathology that has been observed in humans with scrub typhus, which may be correlated with documented clinical manifestations including labored breathing, pulmonary edema, cardiac dysfunction, hepatomegaly, elevations of serum hepatic aminotransferases, and acute renal failure [22,23,24,28,65,66]. The cellular tropism demonstrated by antigen location in pulmonary and systemic endothelial cells, splenic mononuclear phagocytes and cardiac myocytes has been described in scrub typhus autopsies [28]. Relevant animal models, including this sublethal mouse model, advance the understanding of scrub typhus disease kinetics and cellular tropism, which cannot be achieved solely from the limited availability of human patient samples from sublethal infections and lethal outcomes. The ability to study various stages of infection confirms observations of multi-organ involvement and systemic endothelial infection during the acute phase in a sublethal model, substantiating the principal features of scrub typhus disease, which are independent of a lethal outcome. These clinical and pathologic features of scrub typhus should be considered for interpretation of experiments exploring immunological and pathogenic mechanisms of the disease.

Infection of C57Bl/6 mice with *O*. *tsutsugamushi* Gilliam strain, including high, 7.5x10^6^, and mid-dose, 7.5x10^5^, both in excess of the i.v. LD_50_ of 1.25x10^5^ of *O*. *tsutsugamushi* Karp strain, results in sublethal disease evidenced by weight loss and replication of the bacteria in target organs [55]. In the i.v. model, onset of weight loss was preceded by the peak in splenic bacterial burden and coincides with the peak of lung bacterial burden, and these observations were dose-dependent. The peak of lung bacterial load after i.d. inoculation of mice reached the same magnitude as that of the i.v. high and mid-dose; however, it occurred nine days later in the infection at 18 dpi.

Hematologic responses to infection were characterized by increased circulating lymphocytes and neutrophils as well as thrombocytopenia, which have been described in human clinical disease presentation and progression [18,67,68]. IgG antibody titers increase substantially during the first two weeks after onset in human cases [69]. In these murine models, detection of IgG seroconversion by IFA was earliest and most consistent in high dose i.v. infected mice; however, at later time points in the i.d. inoculation model, mice had continually increasing titers. The rate of seroconversion after low dose i.v. challenge was not as consistent as after the higher doses, with a delayed response reaching 100% IgM reactivity by 12 dpi and only 80% IgG seroconversion by 15 dpi. It is unknown whether the i.v. inoculation would trend the same way as the i.d. route, since only the acute disease was characterized after i.v. inoculation.

The antibody response was dominated by the IgG2c isotype, suggesting the importance of Th1 responses in this model. However, IgG1 was detected in all groups except after low dose i.v. infection, which is indicative of Th2 immune response involvement as well. Further experimental time points beyond 15 dpi would need to be assessed after low dose i.v. infection to understand whether a lack of IgG1 reactivity is a difference in immune responses or merely delayed kinetics. We have previously observed a balanced Th1/Th2 response in our sublethal i.d. model of *O*. *tsutsugamushi* Karp infection [56]. In contrast, our lethal i.v. model of *O*. *tsutsugamushi* Karp revealed impairment of select Th2 related immune response molecules [70]. These studies combined suggest that contributions by Th2 responses improve immune homeostasis and result in sublethal outcomes after *Orientia* infection.

In human cases, *Orientia* infects endothelial cells, macrophages and cardiac myocytes in disseminated lethal infection and dendritic cells and macrophages in the human mite inoculation site cutaneous eschar [24,28,29,32]. In contrast to models utilizing non-human primates, no eschar was formed after i.d. inoculation in our murine model [71,72,73]. Location of *Orientia* antigen in the i.v. Gilliam model recapitulates many hallmarks of the human scrub typhus cases. At the peak of bacterial burden after high dose i.v. inoculation, *Orientia* antigen colocalized with endothelial cells of the pulmonary and cardiac microcapillaries, and in splenic and hepatic mononuclear phagocytes. At 18 dpi, the peak of bacterial load after i.d. inoculation, *Orientia* antigen was observed in pulmonary and renal endothelial cells and colocalized with typhus nodules in the brain. The distribution of *Orientia* is directly influenced by the route of inoculation. It has been shown that intramuscular and subcutaneous inoculation results in a disseminated infection involving Kupffer cells and macrophages [57,58]. Intracerebral inoculation and intraperitoneal inoculation have been shown to result in infections initially limited to the central nervous system and peritoneal lining, respectively [55,58]. Dissemination characteristics and the subsequent target cells are relevant to accurately interpret immunologic conclusions, and therefore route is an important consideration in experimental design.

In summary, we present the first comprehensive murine model of consistently sublethal scrub typhus in C57/BL6 mice utilizing the *O*. *tsutsugamushi* Gilliam strain. Infection with *O*. *tsutsugamushi* Gilliam in this model results in dose- and route-dependent kinetics, perceptible clinical signs, and measurable histopathologic lesions, and recapitulates human scrub typhus. These models can be utilized to elucidate the progression and pathogenesis of the majority of scrub typhus cases, which result in untreated non-lethal outcomes [74]. Although the magnitude and persistence of lymphohistiocytic infiltrates during this sublethal infection were unforeseen, we hypothesize that this is indicative of a robust immune response and its capacity to control the infection. This feature of these models highlights the necessity to study the host immune regulation involved in sublethal infection and how it differs from the dysregulation we have reported in the lethal model utilizing *O*. *tsutsugamushi* Karp strain [70]. We will focus future studies on the contributions of immune cell subsets to protection to establish the immunologic foundation necessary for development of an effective vaccine. This model complements the available lethal murine model of scrub typhus and will allow for in-depth mechanistic studies related to cross-protection, lethality, and pathogenesis.

## Supporting information

S1 Fig Dose- and time course-dependent lung histopathology of mice inoculated intravenously or intradermally with *O*. *tsutsugamushi* Gilliam. Scoring system based on intensity of interstitial, perivascular and peribronchial lymphocytic infiltrates and widening of the aveolar septa. Grade 1 (A): Scattered inflammatory cells in focal areas of pulmonary parenchyma and around bronchovascular bundles. Grade 2 (B): Widening of alveolar septa and inflammatory cell infiltrates present multifocally in the pulmonary parenchyma and around bronchovascular bundles. Grade 3 (C): Grade 2 lesions present more diffusely in the pulmonary parenchyma and around bronchovascular bundles. Grade 4 (D): Grade 3 lesions plus areas of atelectasis (100X, bars = 200 μm).
(TIF)

S2 Fig Representative histopathologic findings after inoculation of mice with *O*. *tsutsugamushi* Gilliam strain. Mononuclear cellular infiltrate between cardiac myocytes, 15 dpi-mid-dose i.v. (A), splenic expansion of the periarteriolar lymphoid sheaths and the marginal zone 24 dpi-i.d. (B), liver portal triaditis 12 dpi-mid-dose i.v. (C), and mild meningoencephalitis in the brain at 30 dpi-i.d. (D) (100X, bars = 200 μm).
(TIF)
