# Supplementary figures and images for: Murine models of scrub typhus associated with host control of Orientia tsutsugamushi infection
Source: PLoS Negl Trop Dis. 2017 Mar 10;11(3):e0005453. doi: 10.1371/journal.pntd.0005453 (PMC5362142; doi:10.1371/journal.pntd.0005453)

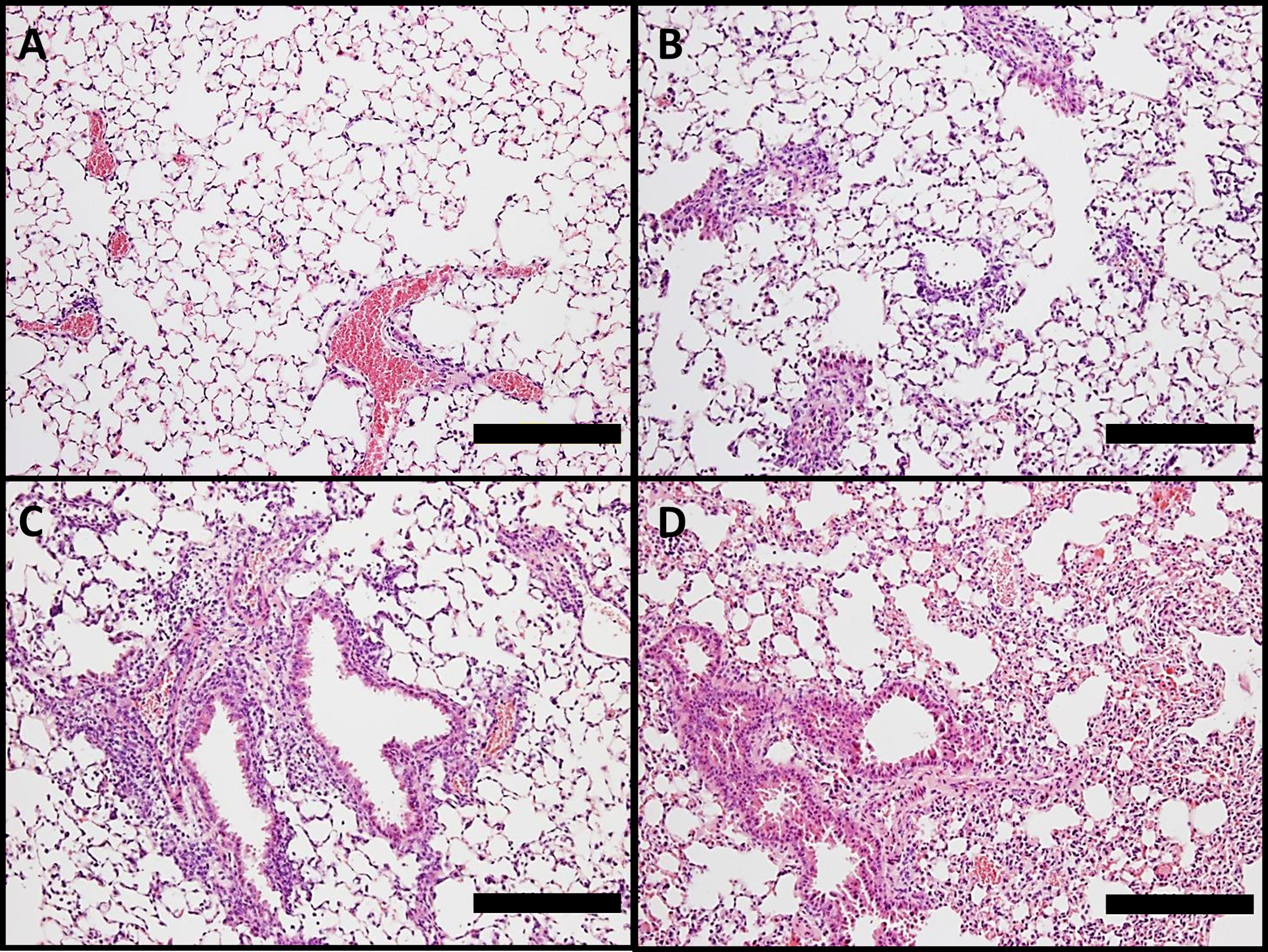

Supplement: S1 Fig — Scoring system based on intensity of interstitial, perivascular and peribronchial lymphocytic infiltrates and widening of the aveolar septa. Grade 1 (A): Scattered inflammatory cells in focal areas of pulmonary parenchyma and around bronchovascular bundles. Grade 2 (B): Widening of alveolar septa and inflammatory cell infiltrates present multifocally in the pulmonary parenchyma and around bronchovascular bundles. Grade 3 (C): Grade 2 lesions present more diffusely in the pulmonary parenchyma and around bronchovascular bundles. Grade 4 (D): Grade 3 lesions plus areas of atelectasis (100X, bars = 200 μm). (TIF) [file pntd.0005453.s001.tif]

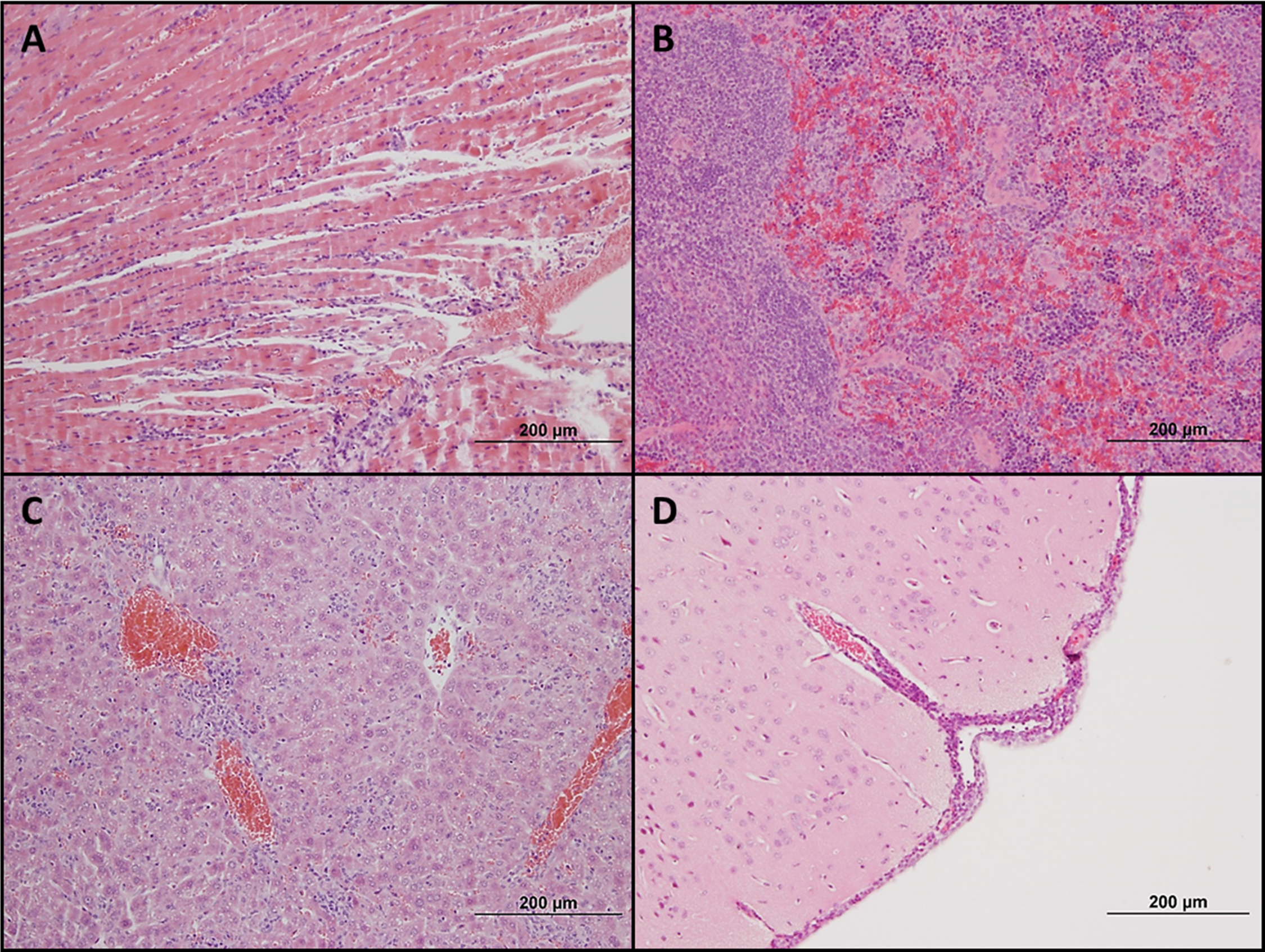

Supplement: S2 Fig — Mononuclear cellular infiltrate between cardiac myocytes, 15 dpi-mid-dose i.v. (A), splenic expansion of the periarteriolar lymphoid sheaths and the marginal zone 24 dpi-i.d. (B), liver portal triaditis 12 dpi-mid-dose i.v. (C), and mild meningoencephalitis in the brain at 30 dpi-i.d. (D) (100X, bars = 200 μm). (TIF) [file pntd.0005453.s002.tif]
